# Recommendations for anesthesia and perioperative management in patients with Ehlers-Danlos syndrome(s)

**DOI:** 10.1186/s13023-014-0109-5

**Published:** 2014-07-23

**Authors:** Thomas Wiesmann, Marco Castori, Fransiska Malfait, Hinnerk Wulf

**Affiliations:** 1 Department of Anesthesiology and Intensive Care Medicine, University Hospital Giessen and Marburg, Campus Marburg, Baldingerstrasse, Marburg, 35037, Germany; 2 Division of Medical Genetics, Department of Molecular Medicine, San Camillo-Forlanini Hospital, Sapienza University, Circonvallazione Gianicolense 87, Rome, 00152, Italy; 3 Ghent University Hospital - UZGent, De Pintelaan 185, Gent, 9000, Belgium

**Keywords:** Ehlers-danlos syndrome, EDS, Anesthesia, Orphan disease, Rare disease, General anesthesia, Epidural anesthesia, Spinal anesthesia, Connective tissue

## Abstract

Ehlers-Danlos syndrome (EDS, ORPHA98249) comprises a group of clinically and genetically heterogeneous heritable connective tissue disorders, chiefly characterized by joint hypermobility and instability, skin texture anomalies, and vascular and soft tissue fragility. As many tissues can be involved, the underlying molecular defect can manifest itself in many organs and with varying degrees of severity, with widespread implications for anesthesia and perioperative management. This review focuses on issues relevant for anesthesia for elective and emergency surgery in EDS. We searched the literature for papers related to all EDS variants; at the moment most of the published data deals with the vascular subtype and, to a lesser extent, classic and hypermobility EDS. Knowledge is fragmented and consists mostly of case reports, small case series and expert opinion. Because EDS patients commonly require surgery, we have summarized some recommendations for general, obstetrical and regional anesthesia, as well as for hemostatic therapy.

## Background

Ehlers-Danlos Syndrome (EDS, Orphanumber: ORPHA98249) is an umbrella term for a growing number of heritable connective tissue disorders, mainly featuring joint hypermobility and instability, skin texture anomalies and vascular and internal organ fragility [[1]–[3]]. The overall incidence is 1:10,000 to 1:25,000 with no ethnic predisposition [[4]], resulting in a presumed number of at least 20,000-50,000 EDS patients in the Northern America. However, the real frequency is probably underestimated due to the general lack of awareness among the various disciplines of such a protean condition, especially for the most atypical presentations. Clinical manifestations range from extremely mild phenotypes to life-threatening complications. The current Villefranche nosology recognizes six major subtypes, comprising classic (corresponding to EDS type I and II of the “old” Berlin nosology), hypermobile (EDS type III), vascular (EDS type IV), kyphoscoliotic (type VIA), arthrochalasia (types VIIA and VIIB) and dermatosparaxis (type VIIC), most of which are linked to mutations in one of the genes encoding for fibrillar collagen proteins or enzymes involved in post-translational modification of these proteins. Recently, several new EDS variants have been defined clinically and genetically. In addition, there has recently been suggested a possible connection between hypermobile EDS and the joint hypermobility syndrome [[5]], a relatively neglected rheumatologic condition recognized by specific diagnostic criteria [[6]]. However, not all researchers and clinicians agree with this view, which may be clarified by future molecular studies [[7]].

For the purposes of this review, we will use the last Villefranche classification. Most EDS subtypes are transmitted as autosomal-dominant or recessive traits; a general overview is presented in Table 1. For a comprehensive overview of genetic and clinical features, there are several published reviews on EDS [[2],[7]]. Surgery is a concern [[8]]. Vascular EDS and the other rarer EDS variants with increased vascular fragility, e.g. classic-like EDS with arterial rupture due to arginine-to-cysteine substitutions in type I collagen [[9]] and kyphoscoliotic EDS are associated with an increased frequency of arterial aneurysms and dissections. Vascular EDS is associated with spontaneous visceral ruptures. Due to the involvement of peri-articular non-ossified connective tissue, many EDS subtypes, especially the hypermobile and arthrochalasis types, may predispose to the need for orthopedic surgery.

**Table 1:** Overview on EDS variants, relevant criteria and genetic background

**Common variant**	**Major criteria**	**Minor criteria**	**Inheritance**	**Causative gene(s)**
**Classic**	Skin hyperextensibility	Smooth, velvety skin	AD	*COL5A1, COL5A2*
	Widened atrophic scars	Molluscoid pseudotumors		
	Joint hypermobility	Subcutaneous spheroids		
		Complications of joint hypermobility		
		Muscle hypotonia, motor delay		
		Easy bruising		
		Manifestations of tissue extensibility and fragility		
		Surgical complications		
		Positive family history		
**Hypermobility**	Hyperextensible and/or	Recurring joint dislocations	AD (?)	Unknown
	smooth, velvety skin	Chronic joint/limb pain		(single reports with mutations in *TNXB* and *COL3A1*)
	Generalized joint hypermobility	Positive family history		
**Vascular**	Thin, translucent skin	Acrogeria	AD	*COL3A1*
	Arterial/intestinal/uterine fragility or rupture	Hypermobility of small joints		
	Extensive bruising	Tendon and muscle rupture		
	Characteristic facial appearance	Talipes equinovarus		
		Early-onset varicose veins		
		Arteriovenous, carotid-cavernous sinus fistula		
		Pneumothorax/pneumohemothorax		
		Gingival recessions		
		Positive family history, sudden death in a close relative		
**Kyphoscoliotic**	Generalized joint hypermobility	Tissue fragility, including atrophic scars	AR	*PLOD1*
	Congenital hypotonia	Easy bruising		
	Congenital and progressive scoliosis	Arterial rupture		
	Scleral fragility and rupture of the ocular globe	Marfanoid habitus		
		Microcornea		
		Osteopenia/porosis		
		Positive family history		
**Arthrochalasis**	Generalized joint hypermobility with recurrent subluxations	Skin hyperextensibility	AD	*COL1A1, COL1A2* (recurrent mutations)
	Congenital bilateral hip dislocation	Tissue fragility, including atrophic scars		
		Easy bruising		
		Hypotonia		
		Kyphoscoliosis		
		Osteopenia/porosis		
**Dermatosparaxis**	Severe skin fragility	Soft, doughy skin texture	AR	*ADAMTS2*
	Sagging, redundant skin	Easy bruising		
		Premature rupture of fetal membranes		
		Large hernias (umbilical, inguinal)		

*Abbreviations:EDS* Ehlers Danlos Syndrome, *AD* autosomal dominat, *AR* autosomal recessive.

## Methods

A literature search was conducted in PubMed and EMBASE for all published literature on the clinical management of EDS. The searches, performed in November 2013, spanned the literature from 1950 to 2013. The following search string was used: Ehlers-Danlos [OR] EDS [AND] anesthesia [OR] anaesthesia [OR] perioperative management. A supplementary search was performed using the references of the selected papers, as well as from personal literature databases (TW, MC and FM). The retrieved literature was screened for information on anesthetic and perioperative management. Interventional studies (experimental or quasi-experimental designs) were defined as those in which an investigator determined the treatment regimen. Observational studies were defined as those in which treatment was decided on the basis of the clinical characteristics of each individual patient. Studies were labeled as case reports or case series when patients were individually described.

This review article does not include data obtained in clinical or laboratory animal studies. Thus, no ethical approval by clinical ethical committees or governmental responsibilities was needed for this article.

## Results

A Pubmed search retrieved 102 articles, and EMBASE 72 articles. Three further articles were obtained from the personal databases of the authors. There was one clinical trial [[10]], the rest were case reports (one or more patients undergoing anesthesia for surgical or obstetrical purposes), retrospective case series or reviews, guideline recommendations or expert opinion. Most case reports and case series deal with vascular EDS, a systematic publication bias towards the relatively rare vascular subtype (EDS subtype IV) (5% of all EDS patients). Furthermore, most retrieved case reports focus on peripartal management (i.e. obstetrical anesthesia). Five retrieved papers offered guidelines for EDS, dealing in part with anaesthetic [[11]], perioperative [[12]–[14]] or peripartal [[15]] topics. Dolan et al. [[16]] published the first recommendations for anesthesia in EDS, and Kuczkowski [[15]] published guidelines for obstetric anesthesia. Castori [[14]] summarized the relevant aspects for surgery and anesthesia in patients with hypermobile EDS. A guideline by Orphanet UK [[12]] focuses on emergencies in vascular EDS. Finally, the authors of this review published a comprehensive peer-reviewed guideline in 2013 at the web-based project “OrphanAnesthesia” (funded by the DGAI, the German society of anesthesiology and Intensive Care medicine) dealing with anesthesia and perioperative issues in patients with EDS of all subtypes.

## Discussion

Literature search did not identify any data with high level of evidence concerning anesthetic issues of EDS; it is mostly single case reports, case series, narrative reviews and expert opinion. We decided to present the published information and discuss its implications, based clinical heuristics, for providing general, regional and local anesthesia and perioperative care. Recommendations for surgical emergency management, obstetrical care and delivery are published elsewhere [[8],[9],[17]]. Main issues regarding perioperative management in patients with EDS are presented in Table 2 and are discussed in the following section.

**Table 2:** Anaesthestic preparations for patients with EDS

**Patient history**	Check for the specific EDS subtype
	Perform thorough bleeding anamnesis
	Patient history with focus on EDS complications
	Check for airway difficulties
**Inform patient**	Inform the patient about relevant Issues of the underlying disorder with regard to planned anaesthesia
	Discuss specific anaesthetic risks for general anaesthesia; discuss regional anaesthesia options with regard to the underlying subtype
**Discuss with surgeon**	Discuss the severity of the respective EDS in each specific patient
	Has your local centre the appropriate facilities and experience for the planned operation AND the underlying EDS?
	Strategies for bleeding therapy in vascular EDS subtypes
	Avoidance of tourniquets, adequate patient positioning
	Avoid ambulatory surgery
	Early mobilization strategy
**Organize**	Cross match blood products, preparation of autotransfusion devices
	Difficult airway management (performed by an expert with adequate devices)
	Prolonged postoperative care facilities
**Inform preoperatively**	Inform all team members including PACU and ward nurses in advance for optimal patient care
**Be aware**	Typical EDS-related emergency-like situations such as difficult airway status, bleeding risks and organ rupture in specific subtypes
**Bleeding prophylaxis**	Use desmopressin in patients with positive bleeding history
**Inform postoperatively**	Inform the patient of any anaesthetic difficulties. Fill out a detailed anaesthesia problem card or medical report to anticipate difficulties in the future.

Classic EDS commonly presents with various degrees of skin fragility and hyperextensibility, defective wound healing, easy bruising and generalized joint hypermobility. Hypermobile EDS is also characterized by minor but often-recognizable skin anomalies and chronic musculoskeletal pain. Vascular EDS features thin, easily bruised, translucent skin, with very marked fragility of blood vessels. The gastro-intestinal tract and gravid uterus, and, less commonly, lungs, spleen and liver, are prone to spontaneous rupture. The natural history and phenotypic spectrum of the other three major variants and all the emerging ones are less well known, but most share tissue fragility and its complications with the more common subtypes. Therefore, carrying out a detailed interview and clinical examination as well as contacting the local/regional reference center is essential in order to estimate the individual anesthetic/procedural risks for each patient. Subtype classification, with particular attention to those variants with increased vascular fragility (especially vascular EDS), should be obtained before surgery.

### Preoperative history and physical examination

Preoperative history and physical examination, general assessment and subtype classification, results of previous clinical genetic counseling, and a standardized bleeding history [[1],[13],[14]] should be taken, as well as assessment of any intubation difficulties. It is helpful to contact the EDS specialist in charge to discuss open questions regarding the specific patient. Skin fragility, with regard to shear forces or use of medical tapes, should be noted. Laboratory results are usually within normal range and mostly not helpful for estimating the bleeding risk. If neuraxial anesthesia is contemplated, scoliosis, previous spinal surgery and other spine pathology should be ruled out. Ineffectiveness of local anesthetics, e.g. during dental procedures in the past, or block failure in previous attempts of local or regional anesthesia should be noted. Muscular weakness and signs of aortic or mitral insufficiency should be sought. A Doppler ultrasound scan might help exclude subclinical cardiac pathology (e.g. non-progressive aortic root dilatation or hemodynamically relevant valve insufficiency).

A key factor is, that patients with EDS are treated in the appropriate facility. Even small, elective surgery might warrant referral to a specialized facility. Medical teams unfamiliar with the specific risks and medical needs of patients with EDS might have a severe impact on morbidity and mortality in this specific population.

### Patient monitoring

Non-invasive monitoring should be performed whenever possible, although some patients are prone to bruising and hematoma formation by repetitive non-invasive blood pressure measurements. On the other hand, invasive blood pressure monitoring runs the risk of vascular wall dissection (mainly for EDS subtypes with vascular fragility); we recommend avoiding invasive measurements whenever possible in non-emergency surgery in vascular EDS. Ultrasound might reduce the risk of repeated vascular puncture.

### Patient positioning

Padding should be used to reduce shear forces and external tissue pressure. The eye should be protected as direct force to the eyeball increases the risk of retinal detachment and globe rupture, especially in kyphoscoliotic EDS and brittle cornea syndromes with increased ocular fragility, and in pathological myopia. One of the authors (TW) has anecdotal knowledge of an iatrogenic retinal detachment by a surgeon’s elbow in a patient during surgery on the contralateral eye. A case report describes a severe stretch injury to the brachial plexus [[18]] due to intraoperative hyper abduction. Adhesive tapes and wound dressing for fixation of devices should be easily removable or avoided if possible because of the risk of severe skin damage; the history should specifically address this issue. This issue is of outstanding importance in EDS with fragile skin such as dermatosparaxis and other subtypes with fragile skin. Patient handling must be performed with maximal reduction of shear forces of the skin as even minor shear forces might result in severe decollement injuries in these patient subgroups. Pre- and postoperative written documentation of the neurological and visual status might be helpful with regard to medicolegal issues in operations with need for extreme patient positioning.

### Airway management & ventilator strategies

In general, mask ventilation, intubation and laryngeal mask insertion are all possible, but care is required with mask ventilation to avoid temporomandibular joint luxation. Repeated intubation attempts may cause bleeding; smaller endotracheal tubes may reduce mucosal damage. Cuff pressure should be checked frequently and kept as low as possible. Airway pressure should be minimised due to the risk of pneumothorax.

Difficult airway management and intubation may occur in many forms of EDS with temporomandibular dysfunction, premature spondylosis, or occipitalatlantoaxial instability [[19]] and should be anticipated with a higher incidence than in general population. This might result in high risk for accidental joint dislocation during intubation attempts or in reduced joint mobility caused by spondylosis resulting in reduced mouth opening. Subclinical cervical spine instability may be present in patients with preserved neck flexibility and temporomandibular joint mobility or occipitoatlantooccipital instability. Care taken here might prevent post-operative complications, such as neck pain and compression-related neurological symptoms. Elective fibreoptic intubation should be considered when difficulties are anticipated.

Laparoscopic surgery might result in higher inspiratory positive pressure to achieve normocapnea during peritoneal inflation. This might result in higher risk for pneumothorax. Peritoneal CO2-inflation might theoretically result in capnothorax. Postoperative chest X-ray or chest sonography might be helpful to rule out this rare condition. Moreover, we recommend that the anesthetist should discuss the use of an open instead of an laparoscopic approach in patients with vascular EDS subtypes with regard to the bleeding risk and better hemostatic options in open approach surgery.

### Circulatory issues

Postural orthostatic tachycardia syndrome (POTS) is sometimes a feature in EDS, especially those with the hypermobile variant (or joint hypermobility syndrome) [[20]]. Preoperative infusion of crystalloid and early use of vasopressors may be helpful [[14]]. The effects of POTS on perioperative care aren’t yet known; adequate postoperative patient monitoring is recommended.

### Crossmatching, bleeding prophylaxis and thromboprophylaxis

Crossmatching adequate amounts of RBCs is advised for patients at risk of bleeding (EDS subtypes with increased vascular fragility, as well as patients with unknown or positive bleeding history). In elective surgery, autologous donation should be discussed. Cell-saving might be advisable for major surgery [[11]]. For details we refer to the review by Castori et al. [[14]]. Although laboratory testing usually shows normal values for INR, PTT and other in-vitro-tests (such as thrombelastometry, e.g. ROTEM™), platelet aggregation abnormalities can be expected in about 26% of patients [[21]]. New measures of platelet function such as PFA-100™ or Multiplate™ show normal values and should not be used routinely. About 90% of EDS patients (all subtypes) bruise easily. In some classic and dermatosparaxis EDS, and EDS subtypes with increased vascular fragility, patients are prone to severe bleeding and hematoma formation. De Paepe and Malfait [[22]] have given a detailed review of bleeding disorders in EDS patients. In acute bleeding (especially in vascular EDS), we recommend an early aggressive hemostatic therapy as well as intraoperative coagulation tests (in laboratory as well as point-of care, e.g. ROTEM™). Desmopressin (DDAVP) improves the bleeding time and reduces transfusion requirements [[23]–[26]] although the exact mechanism is unknown. It increases plasma levels of Factor VIII and von-Willebrand-Factor (vWF). Some authors recommend its use preoperatively in vascular EDS [[8],[11],[27]].

In children with EDS, Mast et al. [[26]] showed that desmopressin decreased the bleeding time to 5.95 ± 2.41 (mean ± SD) min, from 11.26 ± 4.39 min (P < 0,001). A retrospective analysis of 41 surgical procedures in those 26 patients showed no bleeding complications when DDAVP was used compared with 30% of complications when DDAVP was not given preoperatively. As in other bleeding disorders or major surgery, use of prophylactic tranexamic acid before surgery should be contemplated [[28]]. The use of recombinant FVIIa for patients with massive hemorrhagia and coagulopathy featured in two case reports [[28],[29]].

To our knowledge, no recommendations for thromboprophylaxis in patients with EDS are published. This topic should be ruled out on a case-by-case decision with regard to the surgical treatment, individual bleeding risks as well as postoperative mobility in each specific patient.

### Pharmacology

Ehlers-Danlos syndrome is not associated with abnormalities of pharmacokinetics or pharmodynamics, but a detailed history might reveal relevant concurrent disease. General anesthesia can be performed as balanced anesthesia with volatile anesthetics, nitrous oxide or as total intravenous anesthesia (TIVA). Depolarizing (Succinylcholine) as well as non-depolarizing agents are safe. As some EDS patients present with muscular weakness, monitoring of neuromuscular blockade is advised before emergence of the anesthesia. In immobilized patients, the avoidance of depolarizing agents (succinylcholine) is advisable.

### Use of tourniquets

The anesthetist must discuss the benefits and drawbacks with the surgeon if tourniquet is contemplated by the surgeon. There is a high risk of hematoma and compartment syndromes, as well untreatable diffuse bleeding in EDS subtypes with vascular fragility. Some of the present authors have anecdotal knowledge of severe and lethal complications due to the use of tourniquets in elective minor extremity surgery of vascular EDS patients. Diffuse bleeding after use of a tourniquet has resulted in massive compartment bleeding and hemorrhagic shock.

### Central venous catheterization

EDS patients with vascular fragility are at great risks of vascular rupture [[30],[31]]. We recommend the avoidance of central venous access and arterial puncture whenever possible in these subtypes. The use of ultrasound for central venous catheterization is recommended [[32],[33]]. If central lines are needed in EDS patients (high risk surgery, emergencies), ultrasound guidance is mandatory, including visualization of correct wire localization within the blood vessel [[34]]. Use of dilatators should be restricted as they might worsen vascular damage. Jugular vein puncture is preferred to subclavian catheterization with regard to complications such as pneumothorax and bleeding in patients with vascular EDS.

### Regional anesthesia & local anesthesia

There is no clear recommendation for either general or regional/local anesthesia. The Emergency guideline for vascular EDS by Orphanet UK [[35]] strictly recommends avoiding neuraxial blockade, as does Abouleish [[36]] for obstetrical anesthesia. Nevertheless, there are several case reports and case series of successful spinal and epidural anesthesia in patients with vascular EDS, as well as other subtypes. This may be due to an underreporting bias and should not be seen as a general *carte blanche*. Large trials are needed to estimate a potentially increased risk for perforation, nerve injury or hematoma formation in patients with tissue fragility and increased risk of bleeding. This topic should be discussed with the patient with regard to the patient-specific risks (e.g. EDS subtype, bleeding diathesis, spinal pathology, a past history of hematomas or spontaneous organ rupture) and the potential benefits of a neuraxial blockade (early mobilization, pain control, the wish for awareness at caesarean delivery).

A greater rate of postdural puncture headache (PDPH) after neuraxial blockade might be expected, due to tissue fragility, but there are no relevant studies. PDPH can be stopped by an peridural blood patch. Theoretically, this option exists in EDS patients, too.

Moreover, EDS subtypes with tissue fragility are linked to high incidence of spontaneous dural rupture and resulting headache comparable to PDPH. In these specific highly-symptomatic patients, a peridural blood patch might be a minimal-invasive option and might be discussed between neurologist, neurosurgeon and the anesthetist (as most of the anesthetists have regularly experience in performing blood patches due to PDPH).

Neuraxial blockade is of course more complicated in the presence of spinal pathology (scoliosis, kyphosis, spinal stenosis etc.). In addition, isolated or multiple Tarlov cysts ( cerebrospinal-fluid filled perineurial cysts) as a sign of meningeal involvement is a feature of specific EDS subtypes (i.e. classic, hypermobile and kyphoscoliotic). Most Tarlov cysts are located in the S1 to S4 region of the spinal cord and therefore only a relative contraindication for the performance of a spinal anesthesia or thoracal or lumbal epidural anesthesia. However, one of the authors (MC, unpublished data) has observed thoracic Tarlov cysts in hypermobile EDS patients.

Thus, we recommend to avoid neuraxial blockades in patients with vascular EDS based on the lack of clear benefits and the potential risks compared with a planned general anesthesia. Neuraxial blockades in other EDS types are feasible. However, thorough discussion of potential benefits and riscs with the team and the specific patient should be performed. Pre-interventional ultrasound imaging (or use of MRI) might help to rule out relevant spine pathology in some patients who are scheduled for a neuraxial blockade.

As mentioned above, there are few case reports [[37]] of peripheral regional anesthesia in EDS. Nevertheless, because of the frequency of difficult airway status in many EDS subgroups, peripheral regional anesthesia could be indicated in limb surgery. As in neuraxial blockade, thorough history and examination is essential. Tissue scaring can theoretically hamper the spread of local anesthetics. Ultrasound guidance is recommended to reduce the incidence of vascular puncture and obtain better deposition local anesthetic spread. With regard to the bleeding risk, we do not recommend the performance of peripheral nerve blockades in patients with vascular EDS. Peripheral nerve blocks in patients with other EDS subtypes can be advised on a case-by-case shared-decision making policy.

Topical local anesthetics, e.g. EMLA™ (an eutectic mixture of topical lidocaine and prilocaine), or local infiltration anesthesia might be ineffective [[10],[38]], due to tissue scarring, as shown by a clinical study [[39]] using EMLA™ cream in EDS patients and healthy controls. This may be important when using EMLA™ in children to facilitate painless venous puncture.

### Obstetrical anesthesia

There are case series and retrospective analysis [[13],[40]] with parturients for all types of delivery (vaginal, forceps, caesarean section) and for all common types for anesthesia (general, epidural, spinal). There is no agreement on optimal delivery method, as uterine rupture and delayed wound healing may complicate both vaginal delivery and caesarean section. Severe bleeding - especially for patients with vascular fragility - must always be anticipated. Elective caesarean section is best in patients at great risk for complications. Crossmatching of sufficient amounts of RBCs, use of autotranfusion systems and DDAVP as prophylaxis should be discussed.

In hypermobile EDS, episiotomy is associated with to pelvic prolapse [[41]] and should be avoided. Instead of performing episiotomy for vaginal delivery, ceasarean section may be preferred.

There is no general recommendation for the mode of delivery in specific EDS types, which is discussed in detail elsewhere [[15],[41],[42]]. The benefits of neuraxial blockade for obstetrical anaesthesia should be balanced against the increased risks in specific EDS subtypes [[43]] as mentioned above. Some authors favour general anaesthesia in patients with bleeding risks such as in vascular EDS [[12]]. Nevertheless, we recommend to avoid neuraxial regional anesthesia (spinal or peridural anesthesia techniques) in vascular EDS.

Use of patient-controlled i.v. remifentanil instead of neuraxial blockade for vaginal delivery might be a pragmatic approach in patients with high risk for neuraxial complications. In some countries, use of inhalative 50% nitrous oxide is a common alternative for labour analgesia. However, as there is the risk of spontenaous pneumothorax in some patients with EDS, this treatment option should be used only on a case-by-case decision.

### Postoperative care

Little has been written about this topic. Careful patient positioning and mobilisation is advised to reduce the risk of joint luxation as well as reduction of shear forces with regard to the skin fragility. In all EDS subtypes, early mobilization is a essential to prevent loss of strength in the musculoskeletal and cardiovascular systems, particularly in the hypermobile subtype. Patients should be checked thoroughly for development of bleeding and hematomas at the operation site. Patients with POTS should be monitored for signs of cardiovascular instabilitly in the first postoperative hours. Adequate prophylaxis of postoperative nausea and vomiting (PONV) is recommended as spontaneous eosophageal rupture has been reported as a result of vomiting in vascular EDS [[8]].

### Ambulatory anesthesia

All EDS patients should be operated in centres with the relevant expertise and knowledge [[11]]. With suspected or obvious vascular fragility, bleeding (including developing compartment syndromes etc.) should be anticipated. All patients, even after minor surgery, should be monitored for at least 24h.

### Perioperative emergencies

All PACU and ward nurses should be aware of specific acute situations in EDS such as vascular dissection (e.g. aortic dissection, peripheral arteries & veins) caused spontaneously or iatrogenic (especially during angiographic interventions [[8]]). Compartment syndromes can be caused by vascular puncture or rupture due to pressure and shear forces and resulting bleeding. Unstoppable bleeding from the operation site or organ rupture (vascular EDS) may occur. Risk of pneumo- (hemo-) thorax must be anticipated during positive pressure ventilation as well as central venous access. Moreover, spontaneous rupture or rupture after trivial trauma to the bowel, uterus, esophagus or vagina has been reported. This risk is greater in the post-operative period, and may occur at sites remote from the site of surgery. All team members should be aware of these possibilities in the postoperative period to avoid delayed diagnosis when unusual symptoms occur. These events are unusual in patients other than vascular EDS; in this condition, even elective vascular, gastro-intestinal and other surgery should be avoided whenever possible.

### Possible anesthetic complications

Careless patient positioning might enhance the risk for brachial plexus neuropathy or visual loss due to direct pressure to the eye. Skin damage and hematoma formation may occur due to shear forces and insufficient patient padding. Patients with atlantooccipital instability may present airway difficulties and there is a risk of temporomandibular joint luxation during mask ventilation or intubation [[11]]. Scoliosis and other forms of spinal pathology might complicate intubation attempts. In vascular EDS, spontaneous pneumothorax due to mechanical ventilation and upper airway bleeding during repeated intubation attempts may occur [[12]].

As reported above, there may be a predisposition to postdural puncture headache (PDPH), or greater risk of epidural hematoma. Both issues should be discussed with the patient. Nevertheless, this advice comes from expert opinion; there is also a case series of patients with spontaneous CSF leaks with high rates of underlying EDS [[44]]. Published literature of patients with neuraxial blockade (mostly obstetrical anesthesia) shows only one postdural puncture headache (PDPH), and that in a patient with vascular EDS [[40]]. Prospective studies are needed for estimating the risk of PDPH and bleeding complications in neuraxial blockades in EDS patients.

## Conclusion

EDS is challenging for the anesthetist, and there is little evidence-based knowledge.

Therefore, a registry for perioperative complications in patients with EDS might help to improve safety for anesthesia and perioperative medicine in this specific population. However, safe anesthesia can be provided with adequate preparations and personal, organizational and technical resources. The above recommendations must be adequately crosschecked for each patient. The authors recommend prospective acquisition of previous anesthesia charts in any specific patient population to make realistic risk estimation with regard to airway difficulties, bleeding complications and neuraxial techniques and complication rates.

## Competing interests

Parts of these recommendations were presented online as “OrphanAnesthesia Guideline for Anesthesia in Ehlers-Danlos Syndrome” by the authors.

## Authors’ contributions

TW, MC, FC, HW: conception, literature research and analysis, drafting the article. All authors read and approved the final manuscript.
